# Spatial–temporal variations of sediment transport rate and driving factors in Shule River Basin, northwest China

**DOI:** 10.1038/s41598-024-70322-9

**Published:** 2024-08-21

**Authors:** Dongyuan Sun, Yike Wang, Zuirong Niu, Heping Shu, Xingfan Wang, Yanqiang Cui, Yali Ma, Lanzhen Wu

**Affiliations:** College of Water Conservancy and Hydropower Engineering, Gansu Agricultural University, No. 1 Yingmen Village, Anning District, Lanzhou, 730070 Gansu China

**Keywords:** Sediment transport rate, Space–time change, Driving factors, Shule River, Hydrology, Climate-change impacts

## Abstract

The sediment content and transport rate of rivers are crucial indicators reflecting soil erosion, water quality, and water resource management in a region. Studying changes in river sediment transport rates within a basin is essential for evaluating water quality, restoring water ecosystems, and implementing soil and water conservation measures. This study focused on the Shule River Basin and utilized various methods such as moving average, cumulative anomaly, Mann–Kendall mutation test, Mann–Kendall (M–K) trend test, Sen’s slope estimation, Correlation analysis, wavelet analysis, R/S analysis, ARCGIS10.7 interpolation, non-uniformity coefficient, and concentration to analyze data from hydrologic stations at Changmapu (CMP), Panjiazhuang (PJZ), and Dangchengwan (DCW). The research examined the temporal and spatial characteristics of sediment transport rates and identified key driving factors. Findings revealed significant increases in annual sediment transport rates at CMP and PJZ by 12.227 and 4.318 kg/s (10a)^−1^, respectively, while DCW experienced a decrease of 0.677 kg/s (10a)^−1^. The sediment transport rate of the three stations had a sudden change around 1994. The average annual sediment transport rates displayed distinct cycles, with CMP, PJZ, and DCW showing cycles of 51a, 53a, and 29a respectively. Additionally, while CMP and PJZ exhibited a continuous upward trend in sediment transport rates, DCW showed a consistent decline. The annual average sediment transport rates of CMP, PJZ, and DCW were 1305.43 kg/s, 810.06 kg/s, and 247.80 kg/s, respectively. These research findings contribute to enhancing the comprehension of sediment dynamics in the arid region of northwest China and offer a theoretical basis for the restoration and management of ecological environments in similar areas in the future.

## Introduction

River sediment is a complex product of various factors including the evolution of the river itself, climate, underlying surface of the basin, soil and water loss, and human activities. It serves as a crucial indicator reflecting the ecological environment of the basin^1,2^. Primarily originating from surface erosion of the basin and the upstream channel^3,4^, sediment plays a key role in channel erosion and deposition changes, and is a significant factor in evaluating river runoff quality. The sediment transport rate is a vital indicator that objectively reflects changes in soil and water erosion^5^. By examining the size of river sediment, one can assess river erosion and siltation, control soil loss, and evaluate water resources and water ecological quality. River sediment transport is a key metric for quantifying land degradation and soil resource reduction, and is an essential component of the geochemical cycle^6,7^, being particularly sensitive to climate and surface processes. Research on basin sediment transport and deposition is a focal point in the field of water science^8–11^.

The study of sediment transport rate in major rivers around the world has been a hot topic among scholars from various disciplines^12–15^. The sediment transport rate, as the main source of runoff in a basin, plays a crucial role in determining the size of surface runoff. The variation of water and sediment flux depends on many factors such as geographical location, topography, vegetation and climate. The most important factors that determine the variation of river water and sediment flux are mainly attributed to the influence of climate change and human activities in the basin. Climate change can impact sediment transport rates, influencing overall runoff^16–18^. Li et al. conducted a non-parametric trend analysis of long-term data series to identify important trends in water and sand fluxes in various rivers, analyzing their changes under the influence of climate change and human activities^19^. Collins et al. observed that sediment yield tends to be higher in arid and semi-arid climates, while it decreases in humid climates^20^. Zhang et al. reported that the increase in river sediment flux in the source area of the Yangtze River over the past 30 years was primarily due to enhanced runoff erosivity and transport capacity, with increased precipitation contributing to higher sediment content^12^. Dong et al. noted a downward trend in the estuary runoff and sediment load of the Yellow River from 1983 to 2011, with significant inter-annual variations^21^. Tian conducted a study on the effects of climate change and human activities on water and sediment fluxes in the Yellow River, the Yangtze River, and the Pearl River over the past 60 years. The findings revealed that the Yellow River exhibited greater sensitivity to these factors compared to the Yangtze River and the Pearl River^22^. Wang et al. revealed that more than 90% of the sediment in the Yellow River originates from the Loess Plateau^1^, leading to serious flood disasters due to soil erosion^23^. However, research on sediment in arid inland river basins, particularly in the Shule River Basin, remains relatively scarce^24–26^.

Located in an inland river basin within an arid region, the Shule River plays a crucial role in supporting the construction of the Silk Road Economic Belt’s golden section and serves as a vital barrier for ensuring the ecological security of the Qilian Mountains in western China^27^. Scholars have extensively researched the ecological conditions of the basin due to its unique geographical position. For instance, Li et al. conducted a stable isotope study on inland areas, focusing on the Qilian Mountains and Hexi Corridor. Their findings indicated that river runoff and groundwater in the area are primarily consumed by evaporation and recycled within the water cycle, significantly contributing to precipitation with distinct spatial patterns. Ye et al. investigated the impact of climate and underlying surface changes on the upstream runoff of three inland river basins in northwest China from 1960 to 2012^28^. The study revealed that while the annual runoff of all three basins increased, only the Shule River and Heihe River exhibited a significant rise. Additionally, Zhang et al. conducted a detailed analysis of sediment deposition characteristics in the Shule River diversion river, using remote sensing data to explore the sedimentary patterns of the arid differentiated river system^29^. Furthermore, Zhou et al. examined stream generation in semi-arid, glacier-covered, and mountain catchment areas in the upper reaches of the Shule River, suggesting that streams in glacier-covered regions may be diminishing at an accelerated rate^30^.

The research presented in this study offers valuable theoretical insights into the changes in sediment transport rate within the Shule River Basin. It highlights the significant attention given by scholars globally to the sustainable development of this region. The study aims to: (1) analyze the evolution trends and influencing factors of sediment transport in arid inland river basins, such as the Shule River, amidst the backdrop of climate change; (2) Uncover the spatial variations in sediment transport rates within the Shule River Basin; (3) Define the characteristics of sediment transport in arid inland regions, serving as a foundational reference for future studies on managing similar sediment resources and ecological changes. These findings hold substantial importance for combating soil erosion, promoting water resource sustainability in the Shule River Basin, and other arid or semi-arid regions.

## Study area

**Figure 1:**
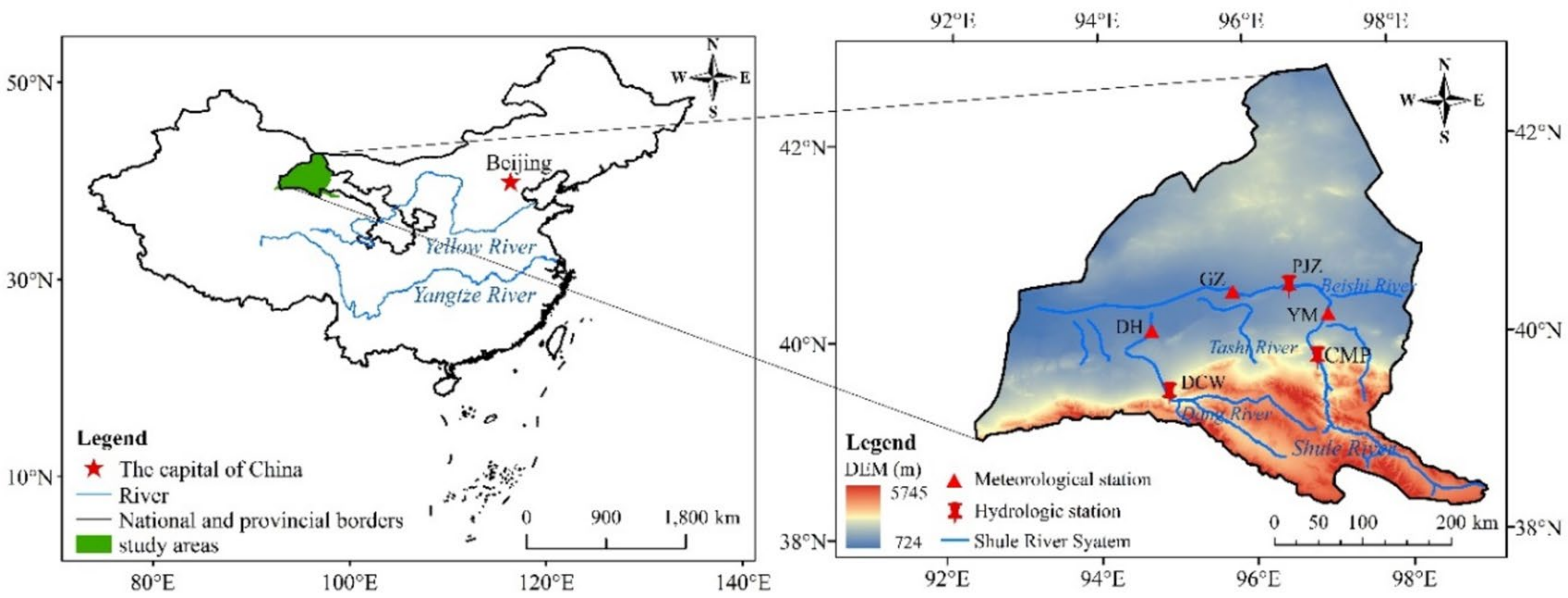
Location of the main hydrological station in the Shule River Basin. Note: The figure made in ARCGIS10.7 (https://desktop.arcgis.com/zh-cn/index.html), the using of natural resources reproduction standard mapping (http://211.159.153.75/index.html). The approval number is GS (2019) 3333, and the boundary of the base map is not modified.

The Shule River Basin, situated in the western part of the Hexi Corridor in Gansu Province, China (Fig. 1), spans a geographical area between 93°10′ to 99° 00′ E and 38° 00′ to 42° 48′ N^31^. Covering a total area of 169,998 km2, the basin features the Changma River as its main stream above Changma Gorge, transitioning to the Shule River below the gorge. Additionally, the basin includes four other rivers: XiaoChangma River, Beishi River, Suanxi River—Yulin River, and Danghe River, with the Danghe River basin being the largest. The region experiences an annual average temperature ranging from 6.98 to 9.82 °C, annual precipitation between 40.2 and 57.5 mm, and annual evaporation from 2577.4 to 2653.2 mm. Known as one of China’s driest areas, the basin faces challenges of minimal precipitation, high evaporation rates, dry and windy conditions, and cold winters. Three hydrographic stations, CMP, PJZ, and DCW, are positioned from east to west within the research area, each providing specific data as outlined in Table 1.

**Table 1:** Information on hydrological stations.

Station name	No. station	site	East longitude	North latitude	Ground ELEVATION/m	Length of data series
CMP	01420800	Gansu	96° 51′	39° 49′	2112.0	1956–2020
PJZ	01421400	Gansu	96° 31′	40° 33′	1340.0	1956–2020
DCW	01423600	Gansu	94° 53′	39° 30′	2176.8	1972–2020

## Materials and methods

### Data sources

The annual and monthly measured sediment discharge data of CMP, PJZ (1956–2020) and DCW (1972–2020) in Shule River Basin are used as the basic data.

The months of March to May, June to August, September to November, and December to February are divided into spring, summer, fall, and winter. The data are from hydrology station of Gansu Province, and the data series is long and representative, which meets the analysis requirements. (https://slt.gansu.gov.cn).

### Methods

The annual and seasonal sediment transport rates of the three stations were analyzed by means of moving average method, cumulative anomaly method, Mann–Kendall (M–K) mutation test, Mann–Kendall (M–K) trend test, Sen’s slope estimation, Correlation analysis, wavelet analysis and R/S analysis, and the mutability, periodicity and persistence of sediment transport rates were analyzed from the aspect of inter-year. The ARCGIS10.7 (https://desktop.arcgis.com/zh-cn/index.html) was used to perform Kriging interpolation on the sediment transport data of three hydrological stations, and then the spatial variation characteristics were analyzed. Using the analysis method of annual distribution of long series runoff, the characteristics of annual variation of sediment transport rate were analyzed by using non-uniformity coefficient and concentration degree.

#### Sliding average and cumulative anomaly method

(1) Moving average method

Several earlier and later values of x_n_ are averaged to obtain a new sequence y_t_. Its calculation formula is as follows^32^:

1 $$y_{t} = \frac{1}{2k + 1}\mathop \sum \limits_{i = - k}^{k} X_{t + i}$$

where y_t_ is the new sequence; k is the sliding average scale; X_t+i_ is the value of the old sequence involved in generating the new sequence. When k = 2, it is 5a moving average; If X_n_ has a trend, select the appropriate k, and its trend can be clearly expressed.

(2) Cumulative anomaly method.

The cumulative anomaly of sediment transport rate is calculated as follows^33^:

2 $$LP_{i} = \mathop \sum \limits_{i}^{N} (R_{i} - \overline{R})$$

where LP_i_ is the accumulated anomaly value of year i; R_i_ is the sediment transport rate in year i. R is the average sediment transport rate for many years. When the cumulative anomaly slope is positive, the sediment transport rate shows an increasing trend. When the cumulative anomaly slope is close to 0, it indicates that the sediment transport rate is stable. When the cumulative anomaly slope is negative, the sediment transport rate shows a decreasing trend.

#### Mann–Kendall mutation analysis

The main calculation steps are as follows: First, assume that a primitive time series variable is X1, X2, …Xn, ri indicates the cumulative number of the ith sample Xi greater than Xj (where 1 ≤ *j* ≤ *i*)^34^. The formula for constructing the statistic Sk is as follows:

3 $$s_{k} = \mathop \sum \limits_{i = 1}^{k} r_{i}$$

4 $$r_{i} = \left\{ {\begin{array}{*{20}c} {0,X_{j} \le X_{i} } \\ {1,X_{j} > X_{i} } \\ \end{array} } \right.,(2 \le k \le n)$$

If the calculated sequence *S*_*k*_ is a random independent time series and follows a normal distribution, then the calculated mean *E(S*_*k*_*)* and variance *Var(S*_*k*_*)* are:

5 $$E\left( {S_{k} } \right) = k\left( {k - 1} \right)/4$$

6 $$Var\left( {S_{K} } \right) = k\left( {k - 1} \right)\left( {2k + 5} \right)/72$$

After it is standardized, the variable is obtained $UF_{k}$:

7 $$UF_{k} = \left[ {S_{k} - E\left( {S_{k} } \right)} \right]/\sqrt {Var\left( {S_{K} } \right)}$$

Under the standard normal distribution, when $UF_{k}$ > 0, it indicates that the series has an upward trend. When $UF_{k}$ < 0, the sequence shows a downward trend. When $UF_{k}$ value exceeded the given confidence level (α = 0.1, α = 0.05, α = 0.01), it indicated that the series showed a significant upward or downward trend.

Arrange the time series X in reverse order *X*′ = {*X*_n_, *X*_n−1_, …, *X*_1_}, repeat the above process to get the trend series $UF_{k}^{\prime }$ of X in reverse order, calculate $UB_{k}$:

8 $$\left\{ {\begin{array}{*{20}c} {UB_{k} = - UF_{k}^{\prime } } \\ {k = n + 1 - k} \\ \end{array} } \right.$$

The two curves of $UF_{k}$ and $UB_{k}$ are drawn in the same M–K test chart, and the change trend of the two curves is observed. When the two curves change outside the confidence interval and do not intersect, it indicates that the sequence has an upward or downward trend. When two curves intersect in the confidence interval, the point at which they intersect is called the abrupt point of the time series.

#### Morlet wavelet analysis

Morlet wavelet analysis^35,36^ in MATLAB is used to calculate the wavelet coefficients and wavelet square differences of various meteorological factors. The main formula calculation steps are as follows:

9 $$\left\{ {\begin{array}{*{20}c} {\int\limits_{R} {\psi \left( t \right)dt = 0} } \\ {\int\limits_{R} {\frac{{\left| {\hat{\psi }\left( \omega \right)^{2} } \right|}}{\left| \omega \right|}d\omega < \infty } } \\ \end{array} } \right.$$

where $\hat{\psi }\left( \omega \right)$ is the spectrum of $\psi \left( t \right)$, $\psi_{a,b} \left( t \right)$ can be obtained by Morlet wavelet analysis:

10 $$\psi_{a,b} \left( t \right) = \left| a \right|^{{ - \frac{1}{2}}} \psi \left( {\frac{t - b}{a}} \right)\;a,b \subset R,(a \ne 0)$$

where ψ is the basic wavelet or parent wavelet, $\psi_{a,b} \left( t \right)$ is a continuous wavelet, *a* is the frequency parameter reflecting the wave, *b* is the time parameter reflecting the displacement, that is, the translation of the wave in time, and R is the set of all real numbers. The continuous form of the function (*t*) wavelet transform is:

11 $$W_{f} \left( {a,b} \right) = \left| a \right|^{{ - \frac{1}{2}}} \mathop \smallint \limits_{R}^{ } f\left( t \right)\overline{\psi }\left( {\frac{t - b}{a}} \right)dt$$

where $W_{f} \left( {a,b} \right)$ is the wavelet coefficient, $\overline{\psi }\left( {\frac{t - b}{a}} \right)$ is the complex conjugate function of $\psi \left( {\frac{t - b}{a}} \right)$, *a* is the time scale factor, and *b* is the displacement factor. In practice, however, climate signals are usually collected discrete. Therefore, *f*(*k*△*t*) is used to indicate (*k* = 1, 2, …, N), △t is the sampling interval. Its discrete expression is:

12 $$W_{f} \left( {a,b} \right) = \left| a \right|^{{ - \frac{1}{2}}} \mathop \sum \limits_{K = 1}^{N} {\text{f}}\left( {{\text{k}}\vartriangle {\text{t}}} \right)\overline{\psi }\left( {\frac{K\vartriangle t - b}{a}} \right)$$

If the squared value of the wavelet coefficient is integrated over the *b* domain, the wavelet square difference can be obtained:

13 $$Var\left( a \right) = \mathop \smallint \limits_{ - \infty }^{ + \infty } \left| {W_{f} \left( {a,b} \right)^{2} } \right|db$$

The contours of the real part of the Morlet wavelet coefficients can reflect the periodic changes of the series at different time scales and its distribution in the time domain. If the real part coefficient is less than 0, it indicates that the time series is in low phase; The denser the contour lines, the stronger the periodic signal of the sequence. Wavelet square difference can be used to determine the main period of the change of different time scale series. The larger the wavelet square difference, the stronger the periodicity of the corresponding time scale.

#### Rescaled range analysis (R/S)

Rescale range analysis (R/S) is a method that can quantitatively reflect the fractal characteristics and long-term memory process of time series. The Hurst index obtained based on R/S analysis can determine whether the time series has obvious trend components and the change trend in the future time period^37^. Therefore, it is widely used in the analysis and study of the change of meteorological data and hydrological elements.

The main principle is to assume that the time series is *Xt* (t = 1, 2, … n), for any positive integer τ ≥ 1, the mean *X̅*(τ) and the cumulative deviation *X*(*t*, τ) are respectively:

14 $$\overline{X}\left( \tau \right) = \frac{1}{\tau }\mathop \sum \limits_{\tau = 1}^{n} X\left( t \right),\left( {\tau = 1,2, \ldots ,n} \right)$$

15 $$X\left( {t,\tau } \right) = \mathop \sum \limits_{t = 1}^{\tau } ( X\left( t \right) - \overline{X}\left( \tau \right)) \left( {1 \le {\text{t}} \le {\uptau }} \right)$$

The expressions for calculating range *R(τ)* and standard deviation *S(τ)* are as follows:

16 $$R\left( \tau \right) = {}_{{1 \le {\text{t}} \le {\uptau }}}^{max} X\left( {t,\tau } \right) - {}_{{1 \le {\text{t}} \le {\uptau }}}^{min} X\left( {t,\tau } \right)$$

17 $$S\left( \tau \right) = \sqrt {\frac{1}{\tau }\mathop \sum \limits_{t = 1}^{\tau } ( X\left( t \right) - \overline{X}\left( \tau \right))^{2} }$$

Therefore, the final Hurst index H can be obtained through linear simulation by least square method, and the specific calculation process is as follows:

18 $$R\left( \tau \right)/ S\left( \tau \right) = \left( {\alpha \tau } \right)^{H}$$

19 $$\ln ( R\left( \tau \right)/ S\left( \tau \right)) = H\ln \tau + H\ln \alpha$$

20 $$H = \ln ( R\left( \tau \right)/ S\left( \tau \right))/\left( { \ln \tau + \ln \alpha } \right)$$

Through the above calculation, it can be seen that the Hurst index has three different forms of variation: when 0.5 < H < 1, it indicates that the future trend of the time series is consistent with the past trend, and the closer the H value is to 1, the greater the consistency; When H = 0.5, it indicates that the time series is considered random, meaning that future trends are independent of past trends; When 0 < H < 0.5, it indicates that the future trend of the time series is opposite to the past trend, and the closer the H value is to 0, the stronger the anti-persistence.

#### Heterogeneity and concentration

At present, there are few analysis methods for the annual variation of long-series sediment transport rate. This paper uses the analysis method of annual distribution of long-series runoff to analyze the annual variation characteristics of sediment transport rate at CMP, PJZ and DCW stations in Shule River Basin in recent decades. The specific principle and calculation process are shown in the literature.

1. Non-uniformity coefficient (Cv)

From the point of view of hydrological analysis, it indicates some characteristics of sediment distribution during the year^38^. The formula for calculating the annual distribution uneven coefficient of sediment transport rate is as follows:

21 $$C_{v} = \frac{{\left( {\mathop \sum \nolimits_{t = 1}^{n} r_{t} - nr_{o} } \right)}}{{12r_{o} }}$$

In this context, let $r_{t}$ represent the monthly average sediment transport rate that exceeds the annual average sediment transport rate, while $r_{0}$ denotes the average annual sediment transport rate. Additionally, n refers to the number of months during which the sediment transport rate surpasses the annual average.

(2) Concentration (RCD)

Concentration degree and concentration period method^39^, calculated as follows:

22 $$RCD_{year} = \frac{{\sqrt {{ }R_{x}^{2} + R_{y}^{2} } }}{{R_{year} }}$$

23 $$RCP_{year} = arctan\frac{{R_{x} }}{{R_{y} }}$$

24 $$R_{x} = \mathop \sum \limits_{i = 1}^{12} r_{i} \sin \theta_{i} ,\;R_{y} = \mathop \sum \limits_{i = 1}^{12} r_{i} \cos {\uptheta }_{{\text{i}}}$$

where RCD_year_ is the concentration of annual sediment transport rate; RCP_year_ is the concentrated period of annual sediment transport rate. R_year_ represents the annual sediment transport rate, while R_x_ and R_y_ denote the horizontal and vertical components, respectively, derived from the summation of the components over a period of 12 months. Here, $r_{i}$ signifies the sediment transport rate for the first month, and ${\uptheta }_{{\text{i}}}$ indicates the vector angle of the sediment transport rate during the i-th month. The larger the RCD_year_, the more dispersed the sediment transport rate, and the smaller the RCD_year_, the more concentrated the sediment transport rate. RCP_year_ indicates the time when the maximum sediment transport rate occurs in a year. Table 2 shows the Angle values contained in each month.

**Table 2:** RCP_year_ calculates the included and representative angle values for each month.

Month	Jan.	Feb.	Mar.	Apr.	May.	Jun.	Jul.	Aug.	Sep.	Oct.	Nov.	Dec.
Inclusion angle/(°)	345–15	15–45	45–75	75–105	105–135	135–165	165–195	195–225	225–255	255–285	285–315	315–345
Selected angle /(°)	0	30	60	90	120	150	180	210	240	270	300	330

#### Sen’s slope estimation method

Sen's slope method is a robust non-parametric calculation method used to describe the trend of long-term data series. It has the advantages of simple calculation, strong noise resistance, and is not easily affected by outliers, so it is widely used in meteorology, hydrology and other studies^40^. For the time series Xt = (X_1_, X_2_, X_3_, …, Xn), the specific calculation process of Sen's slope estimation method is as follows:

25 $$Q_{i} = \frac{{x_{j} - x_{k} }}{j - k},\;\left( {i = 1, \ldots N} \right)$$

where, $x_{j}$ and $x_{k}$ are the time series values of the JTH and KTH samples respectively (J > K), $N = \frac{{n\left( {n - 1} \right)}}{2}$. The same time, N $Q_{i}$ values are arranged from small to large, then the median Sen's slope is estimated as:

26 $$Q_{med} = \left\{ {\begin{array}{*{20}c} {Q\left[ {\frac{N + 1}{2}} \right], \;\;N\, is\, odd} \\ {\frac{{Q\left[ \frac{N}{2} \right] + Q\left[ {\frac{N + 2}{2}} \right]}}{2}, \;\;N\, is\, even} \\ \end{array} } \right.$$

Slope $Q_{med}$ represents the average rate of change of the series and the trend of the time series. When $Q_{med}$ > 0, it indicates that the series is on the rise. When $Q_{med}$ < 0, the sequence showed a downward trend.

#### Mann–Kendall trend test method

Mann–Kendall test (Mann–Kendall method, referred to as M–K method) is a non-parametric statistical test method recommended by the World Meteorological Organization, which is widely used to analyze the trend of time series of environmental data^41^. It is assumed that a random time sequence $x_{i}$ (i = 1, 2, … n) Random independent, define the calculation formula of statistical variable S as:

27 $$S = \mathop \sum \limits_{i = 1}^{n - 1} \mathop \sum \limits_{j = i + 1}^{n} sin(x_{j} - x_{i} )$$

28 $$sin(x_{j} - x_{i} ) = \left\{ {\begin{array}{*{20}c} { - 1 \:\:X_{j} < X_{i} } \\ {0 \:\:\:\;X_{j} = X_{i} } \\ {1\:\:\:\; X_{j} > X_{i} } \\ \end{array} } \right.$$

where n is the length of time series; $X_{j}$ and $X_{i}$ are the corresponding data of time series; $sin\left( {x_{j} - x_{i} } \right)$ is the corresponding sign function. When n ≥ 10, the random sequence S_i_ (i = 1, 2, … n) approximately follows a normal distribution, and the expectation E(S) and variance Var(S) of S are:

29 $$E\left( S \right) = 0$$

30 $$Var\left( S \right) = n\left( {n - 1} \right)\left( {2n + 5} \right)/18$$

M–K statistical test value Z is expressed as:

31 $$Z = \left\{ {\begin{array}{*{20}c} {\frac{S - 1}{{\sqrt {Var\left( S \right)} }}\;\;\; S > 0} \\ {0\;\;\;\;\;\;\;\;\;\;\;\;\: S = 0} \\ {\frac{S + 1}{{\sqrt {Var\left( S \right)} }} \;\;\;S < 0} \\ \end{array} } \right.$$

The obtained standard value Z is compared with the standard normal distributions of the confidence levels (α = 0.1, α = 0.05, α = 0.01). If |*Z*|≥ *Z*1 − *a*⁄2, it indicates that the null hypothesis is invalid and Z follows the normal distribution, that is, the time series has a clear upward or downward trend. When Z > 0, it indicates that the sequence has an upward trend. When Z < 0, the sequence shows a decreasing trend. At the same time, when the absolute value of Z is greater than or equal to 1.64, 1.96 and 2.58, it indicates that the time series has passed the significance test of the confidence level of 90%, 95% and 99%, respectively.

#### Correlation analysis

Correlation analysis refers to the analysis of two or more variable elements with correlation, so as to measure the degree of correlation between two variable factors^42^. In this study, the correlation coefficient r was calculated by using Excel 2022 software and the scatter plot was drawn. At the same time, the effects of rainfall, runoff, surface evaporation and air temperature on the sediment transport rate in Shule River Basin were analyzed by combining the correlation coefficient classification (Table 3). The main calculation formula is as follows:

32 $$r = \frac{{\mathop \sum \nolimits_{i = 1}^{n} (x_{i} - \overline{x})\left( {t_{i} - \overline{t}} \right)}}{{\sqrt {\mathop \sum \nolimits_{i = 1}^{n} \left( {x_{i} - \overline{x}} \right)^{2} \mathop \sum \nolimits_{i = 1}^{n} \left( {t_{i} - \overline{t}} \right)^{2} } }}$$

where, *r* is the correlation coefficient corresponding to *x* and *t*, and the correlation coefficient is between [− 1, 1], and *x* and *t* are the average values of *x* and *t*. The closer |*r*| is to 0, the smaller the linear correlation between the variable *x*_i_ and *t*_i_; The closer |*r*| is to 1, the greater the linear correlation between the variable xi and ti. When *r* > 0, it indicates a positive correlation between the two factors; when *r* < 0, it indicates a negative correlation between the two factors.

**Table 3:** Correlation coefficient grading scale.

Level	Absolute value of correlation coefficient	Degree of correlation
I	0–0.3	Weak correlation
II	0.3–0.5	Low correlation
III	0.5–0.8	Moderately correlated
IV	0.8–1	Highly correlated

## Results

### Annual variation characteristics of sediment transport rate

**Figure 2:**
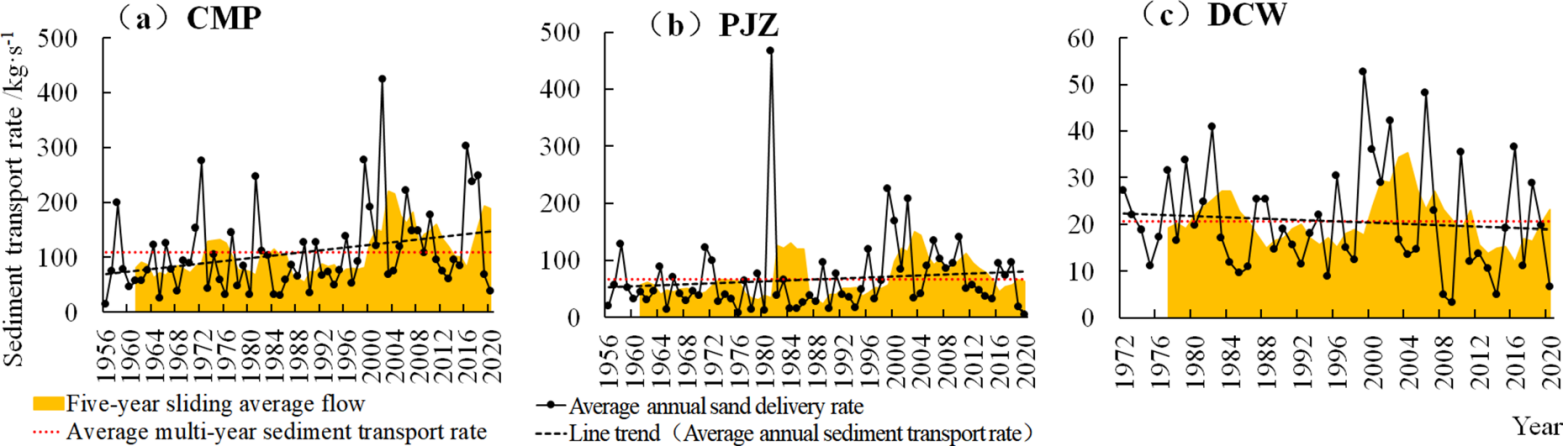
Variation trend of sediment transport rate in Shule River basin.

The annual average sediment transport rates of CMP and PJZ (Fig. 2a,b) from 1956 to 2020 were 108.786 kg/s and 67.505 kg/s, showing an overall increasing trend. The annual sediment transport rate of CMP increased at a rate of 12.227 kg/s/10a and 79.48 kg/s within 65a, showing a significant increasing trend (|Z|> 1.96). The 5a moving average curve of the annual sediment transport rate of CMP shows a three-peak pattern. The annual sediment transport rate of PJZ increased at a rate of 4.318 kg/s/10a, and increased by 28.07 kg/s within 65a, with no significant increasing trend (|Z|< 1.96). The 5a moving average curve of the annual sediment transport rate of PJZ shows a bimodal pattern.

DCW (Fig. 2c) shows that the annual average sediment transport rate from 1972 to 2020 was 20.65 kg/s, and the annual sediment transport rate decreased at a rate of 0.677 kg/s/10a, and decreased by 3.32 kg/s within 49a, with a significant downward trend (|Z|> 1.96). The 5a moving average curve of DCW annual sediment transport rate showed a bimodal pattern, and the peak years were 1984 and 2004 respectively.

In general, the 5a sliding average of sediment transport rates of CMP, PJZ and DCW in the Shule River Basin has similar changes (Fig. 2), showing an annual fluctuation process of “up-down-up-down-up” in recent decades. However, the sediment transport rate of CMP and PJZ showed an increasing trend, and the rising rate of CMP was higher than that of PJZ, while the sediment transport rate of DCW showed a slow decreasing trend.

**Figure 3:**
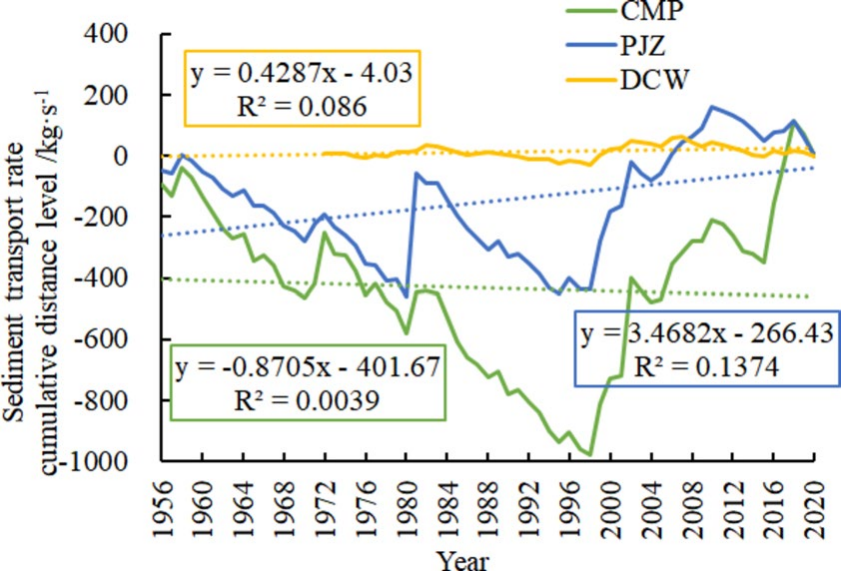
Cumulative spatial variation of sand delivery rates in the Shule River Basin.

The cumulative anomaly shapes of CMP and PJZ were similar (Fig. 3), both of which experienced a process of “ up-down-up-down-up”, with the rising turning points around 1982 and 1998 respectively, and the falling breaking points around 1984. However, the DCW cumulative anomaly curve has a gentle shape and small fluctuation range. The cumulative anomaly slope of CMP − 0.8705 is less than 0, indicating that the sediment transport rate has a slow decreasing trend. The cumulative anomaly slope of PJZ 3.4682 is positive, and the sediment transport rate shows an increasing trend. The cumulative anomaly slope of DCW 0.4287 is positive, indicating that the sediment transport rate shows a slow upward trend.

### Characteristics of seasonal variation of sediment transport rate

**Figure 4:**
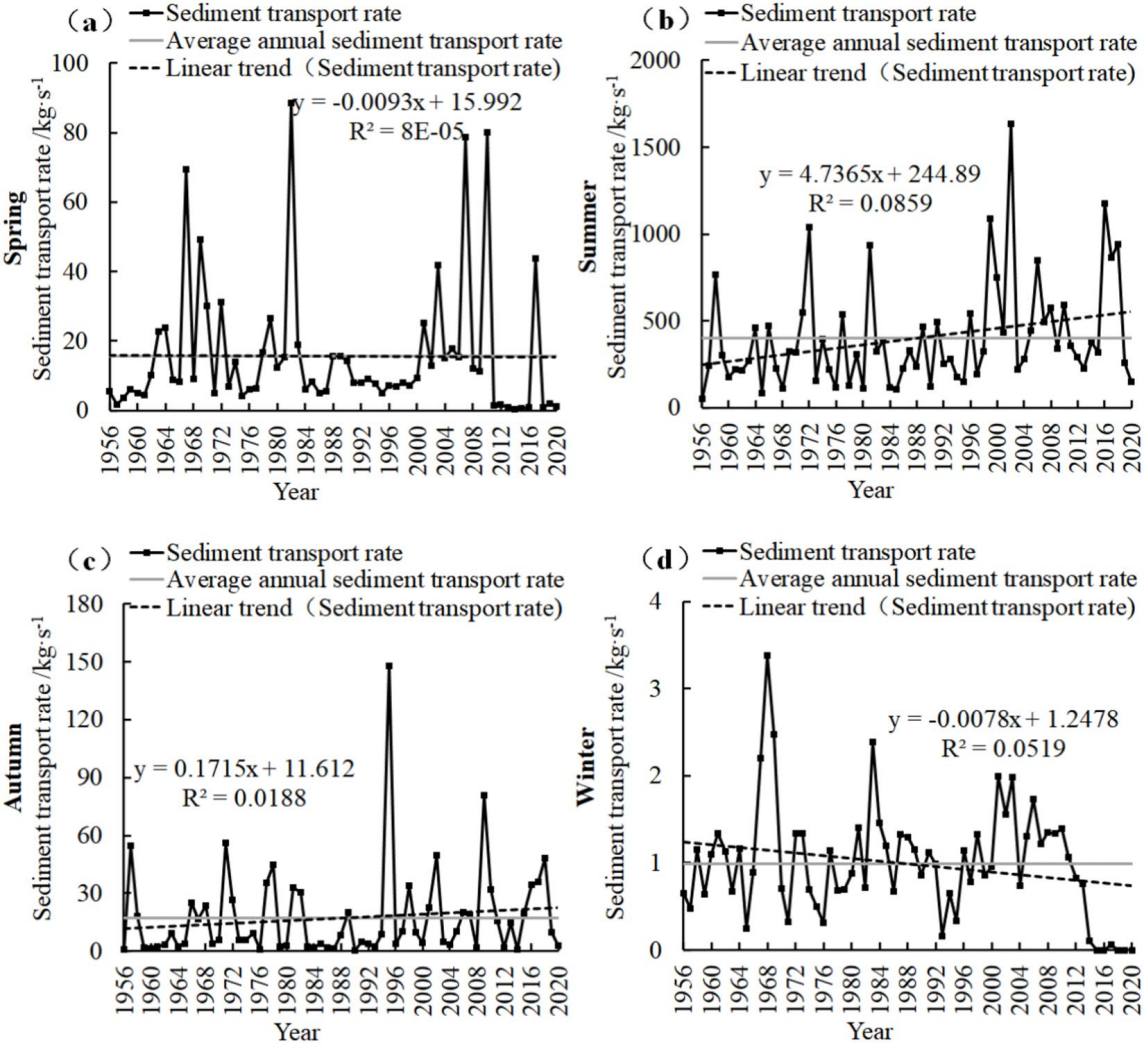
Seasonal sand transport rate change map of CMP Hydrological Station.

CMP (Fig. 4) spring sediment transport rate decreased by 0.61 kg/s in recent 65 years, with a linear decrease of 0.093 kg/s/10a. In winter, the decrease was 0.51 kg/s, and the linear decrease was 0.078 kg/s/10a. Summer and autumn increased by 307.87 kg/s and 11.15 kg/s, respectively, and the linear increases were 47.365 kg/s/10a and 1.715 kg/s/10a, respectively.

**Figure 5:**
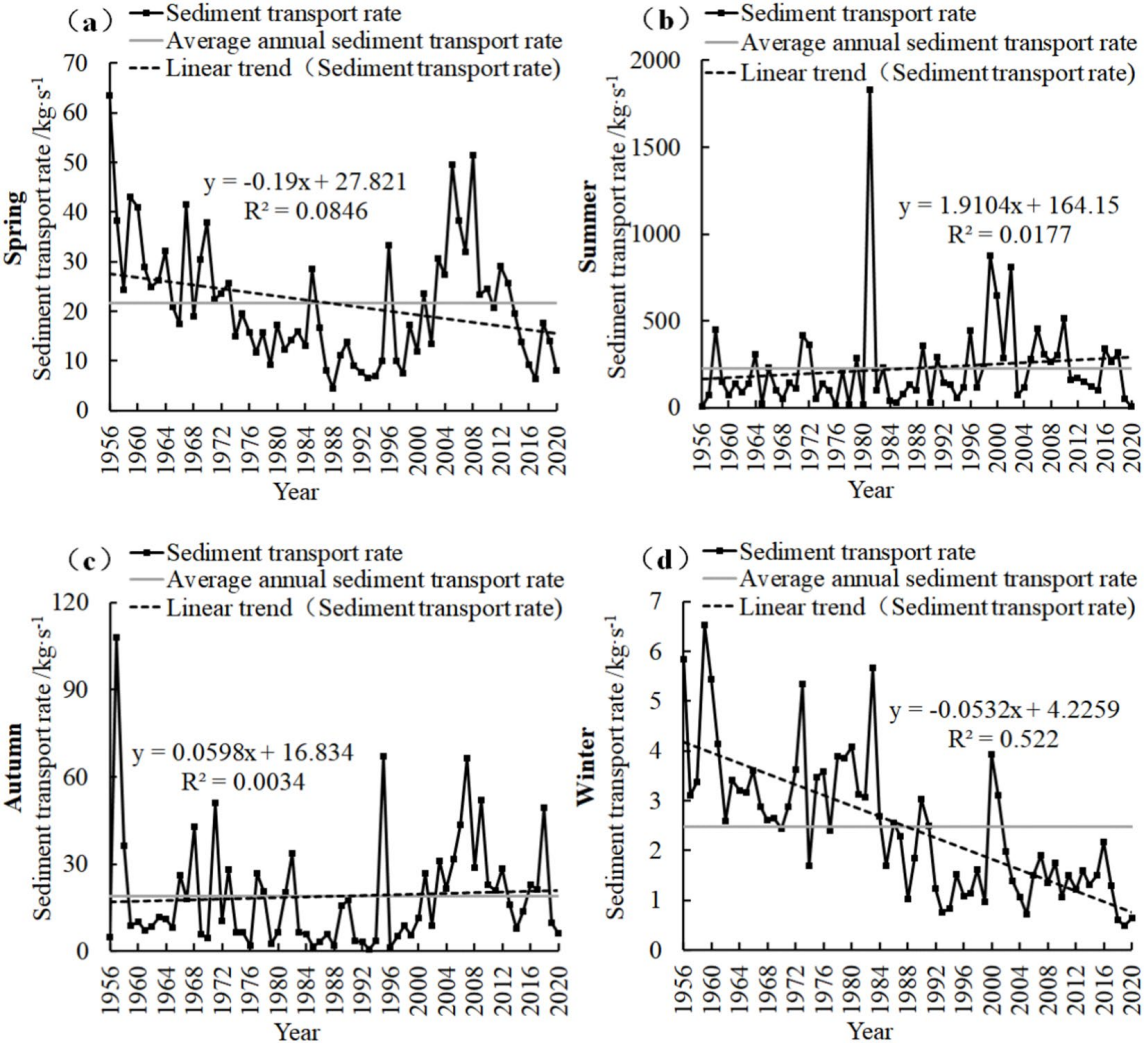
Seasonal sand transport rate change map of PJZ Hydrological Station.

In the past 65 years, the spring sediment transport rate of PJZ (Fig. 5) decreased by − 23.47 kg/s, with a linear decrease of − 1.9 kg/s/10a. In winter, the decrease was − 3.458 kg/s, and the linear decrease was − 0.532 kg/s/10a. Summer and autumn increased by 124.18 kg/s and 3.89 kg/s, respectively, with linear increases of 19.104 kg/s/10a and 0.598 kg/s/10a, respectively.

**Figure 6:**
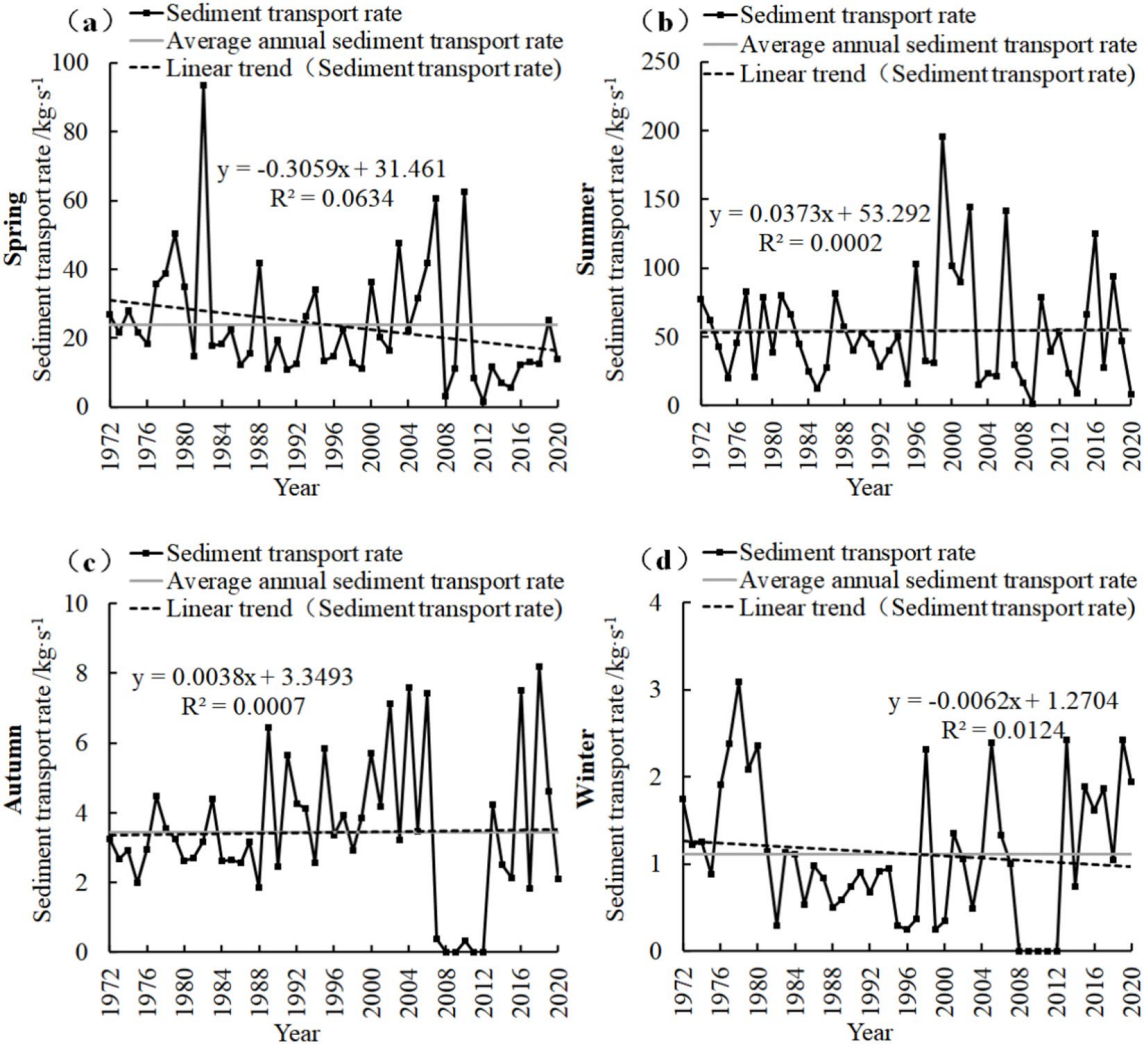
Seasonal sand transport rate change map of DCW Hydrological Station.

In DCW (Fig. 6), the sediment transport rate in spring (Fig. 6a) decreased by − 15 kg/s in recent 49 years, with a linear decrease of − 3.059 kg/s/10a. In winter, the decrease was − 0.3 kg/s, and the linear decrease was − 0.062 kg/s/10a. Summer and autumn increased by 1.83 kg/s and 0.19 kg/s, respectively, and the linear increases were 0.373 kg/s/10a and 0.038 kg/s/10a, respectively.

The sediment transport rates at the three stations (Figs. 4, 5 and 6) exhibited a fluctuating downward trend during spring, with DCW demonstrating the most significant variation, followed by PJZ, while CMP showed the least change. In summer, all stations experienced an increasing trend in sediment transport rates, with CMP showing the most pronounced increase, followed by PJZ, and DCW exhibiting the least change. Autumn also saw an increase in sediment transport rates across all stations, with PJZ again showing the most significant change, followed by CMP, and DCW showing the least. Conversely, winter was characterized by a downward trend in sediment transport rates, with PJZ displaying the most substantial change, followed by CMP, and DCW showing the least variation. These results indicate that the increase in sediment transport rates during summer contributes most significantly to the overall increase in average sediment transport rates, whereas the increase during spring contributes the least.

### Mutation characteristics of sediment transport rate

**Figure 7:**
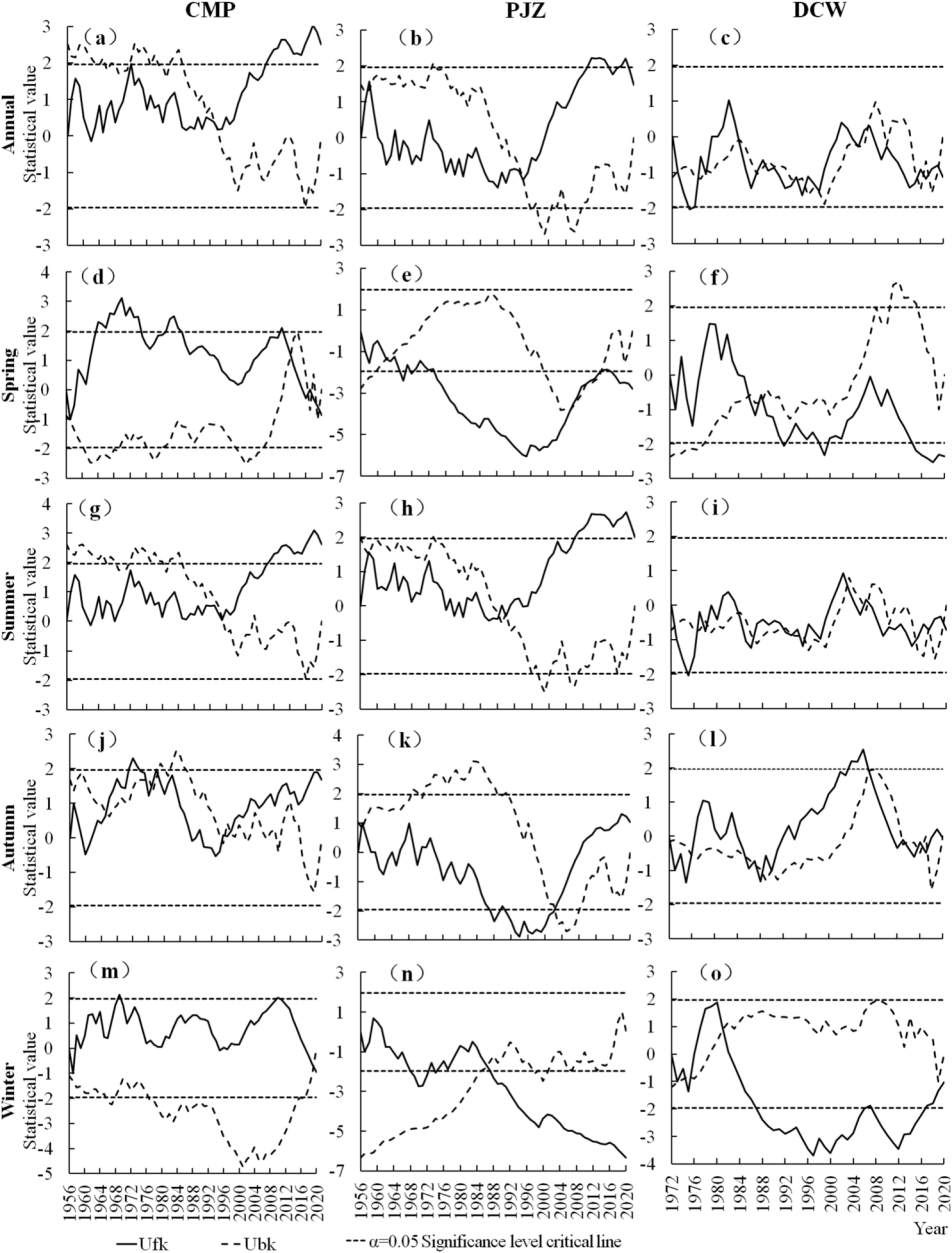
Statistical curve of abrupt change analysis of sediment transport rate in Shule River Basin.

The M–K statistical curve of sediment transport rate of each station in Shule River Basin (Fig. 7), Ufk and Ubk. If Ufk or Ubk value is greater than 0, it indicates that the sediment transport rate series shows an upward trend, and vice versa. The dashed line in the figure represents the critical value at the significant level of α = 0.05. When the statistical curve exceeds the critical line, it indicates a significant upward or downward trend.If two statistical curves, Ufk and Ubk, intersect at the critical boundary, then the intersection is the time when the mutation begins.

The annual average sediment transport rate in the Shule River Basin (Fig. 7a) demonstrates a fluctuating growth trend. After 2006, the UF curve exceeded the significance threshold of 0.05, indicating a significant upward trend in sediment transport rates. The UF and UB curves intersected in 1994, remaining within the 0.05 significance level threshold, suggesting that mutations occurred in that year. The spring sediment transport rate (Fig. 7d) exhibited a downward-upward-downward fluctuation trend, with abrupt changes likely occurring around 2013, and 2019. The summer sediment transport rate (Fig. 7g) showed an overall fluctuating upward trend, with a significant change potentially occurring around 1995. The autumn sediment transport rate (Fig. 7j) also displayed a fluctuating upward trend, with mutations likely occurring around 1965, 1977, and 1996. Finally, the winter sediment transport rate (Fig. 7m) demonstrated a trend of decrease, increase, and subsequent decrease, with inflection points of increase and decrease identified at 1958 and 2017, respectively. A mutation is anticipated to occur around 2020.

The average annual sediment transport in PJZ (Fig. 7b) exhibited an initial decrease followed by an increase, with a significant upward trend observed from 2009 to 2014, as well as in 2017 and 2018, resulting in an overall weak upward trend. A notable mutation may have occurred in 1994. In spring (Fig. 7e), the sediment transport rate demonstrated a downward trend, with potential mutations around 1962 or 2013. During summer, the sediment transport rate displayed an overall fluctuating upward trend (Fig. 7h), with a marked increase after 2014; a mutation likely occurred around 1988. In autumn (Fig. 7k), the sediment transport rate generally exhibited a fluctuating downward trend, with a pronounced decline from 1987 to 2002, suggesting a mutation around 2003. In winter, the sediment transport rate (Fig. 7n) showed a general trend of fluctuation and decline, with significant reductions noted from 1968 to 1973 and after 1987, indicating a probable mutation around 1987.

The average annual sediment transport rate of DCW (Fig. 7c) showed an upward trend in a few years, but did not break the 95% confidence level, and the overall trend was still fluctuating and declining. The UF curve broke through the 0.05 significance level threshold in 1975, and the sediment transport rate decreased significantly. Abrupt changes may occur around 1978, 1987, 1994, 2005 and 2018. The sediment transport rate showed a fluctuating downward trend in spring (Fig. 7f). The sediment transport rate showed a general trend of fluctuation and decline in summer (Fig. 7i). The sediment transport rate in autumn (Fig. 7l) showed an upward trend in 1977–1984 and 1995–2011, and a downward trend in other years. In winter (Fig. 7o), the sediment transport rate generally shows a fluctuating downward trend.

In summary, the sediment transport rates of CMP, PJZ and DCW show different degrees of mutation and different time mutation points throughout the year and in different seasons. However, the annual sediment transport rate of the three stations had a sudden change around 1994.

### Periodic characteristics of sediment transport rate

**Figure 8:**
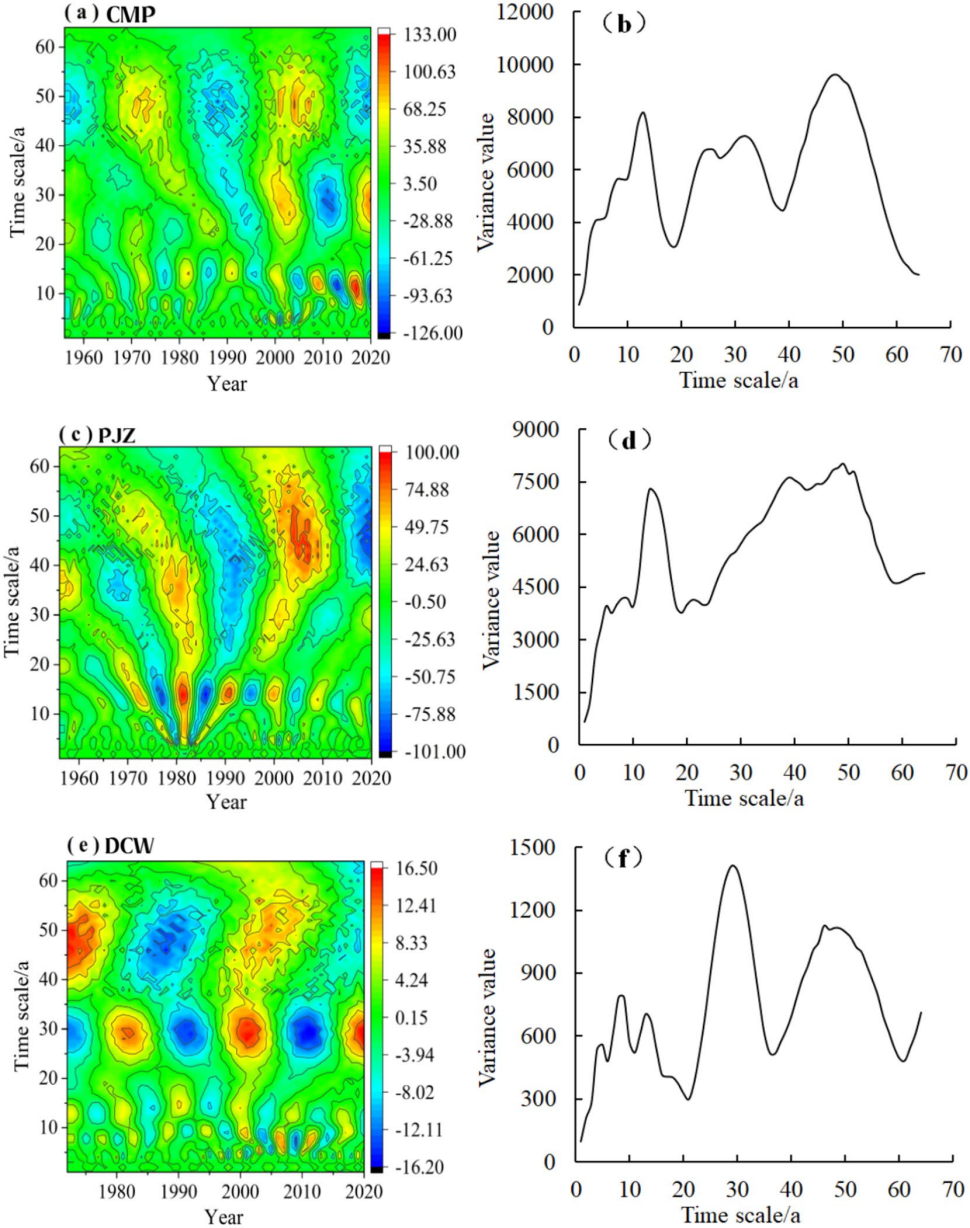
Real map of wavelet coefficient and wavelet variance plot of Basin for the whole year.

The real part time–frequency graph and wavelet square variance graph of the annual sediment transport rate transformation coefficients of three stations in Shule River Basin were calculated by using wavelet analysis method (Fig. 8). The real part of the annual sediment transport rate of CMP (Fig. 8a,b) mainly has three characteristic time scales: 47–55a, 27–33a and 10–15a. PJZ (Fig. 8c,d) mainly has three characteristic time scales: 50–55a, 1318a and 3–8a. DCW (Fig. 8e,f) mainly has three characteristic time scales: 26–32a, 12–15a, and 5–11a. The first main period of the three stations is 51a, 53a and 29a, respectively. The wavelet square variogram indicates that there are 3, 3, and 4 peaks in the annual sediment transport rate for the three stations, respectively.

### Sustainability characteristics of sediment transport rate

**Figure 9:**
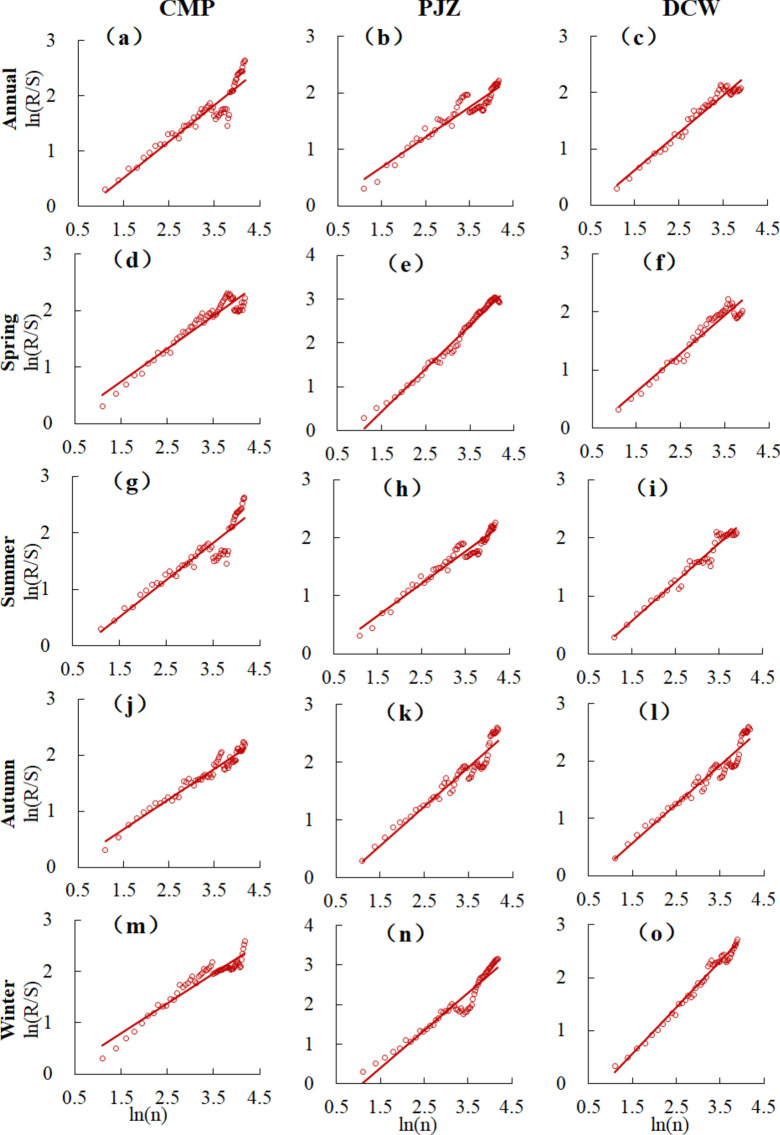
Continuous change chart.

When 0.5 < H < 1, the closer the H value is to 1, the greater the consistency; When H = 0.5, the future trend has nothing to do with the past trend; When 0 < H < 0.5, the closer the H value is to 0, the stronger the anti-persistence is. According to the Hurst index calculations for the annual and seasonal sediment transport rates at CMP (Table 4, Fig. 9), the Hurst indices are 0.6609, 0.5824, 0.6552, 0.5418, and 0.5828, respectively. All these values exceed 0.5, suggesting that future sediment transport will follow a similar trend to that observed over the past 65 a, indicating characteristics of persistence. The Hurst indices for the annual and seasonal sediment transport rates at PJZ are 0.5314, 0.9811, 0.5540, 0.6789, and 0.9480, all of which are also greater than 0.5, demonstrating continuous growth characteristics. Notably, the Hurst index in spring is the highest at 0.9811, indicating strong continuity. For DCW, the Hurst indices are 0.6672, 0.6563, 0.6622, 0.8678, and 0.8718, all exceeding 0.5, which implies that future sediment transport will mirror the trends of the past 49a, exhibiting a continuous decreasing feature.

**Table 4:** Results of continuous analysis of sediment transport rate throughout.

Station	Annual	Spring	Summer	Autumn	Winter
CMP	0.6609	0.5824	0.6552	0.5418	0.5828
PJZ	0.5314	0.9811	0.5540	0.6789	0.9480
DCW	0.6672	0.6563	0.6622	0.8678	0.8718

Combined with the Hurst index calculation results of the annual and four season sediment transport rates of the three stations (Table 4), it can be seen that the Hurst index of the annual and four season sediment transport rates of the three stations is greater than 0.5, which indicates that the future sediment transport rates of CMP and PJZ stations will have the same changing trend as in the past 65 years, showing an increasing trend. The future sediment transport rate of DCW will be the same as the change trend of the past 49a, showing a decreasing trend, and the overall characteristics are continuous.

### Variation characteristics of non-uniformity coefficient and concentration of sediment transport rate

The average coefficient of non-uniformity in Shule River Basin is 0.15–0.35, and the average concentration is 0.22–0.52. The uneven distribution coefficient and concentration degree of sediment transport rate at the three stations show an increasing trend. In CMP and PJZ 65a, 41a and 40a were above the mean value, respectively, indicating that the distribution of sediment transport rate is more centralized. The concentration of sediment transport rate in DCW is 23a higher than the average, and the distribution of sediment transport rate is scattered.

**Figure 10:**
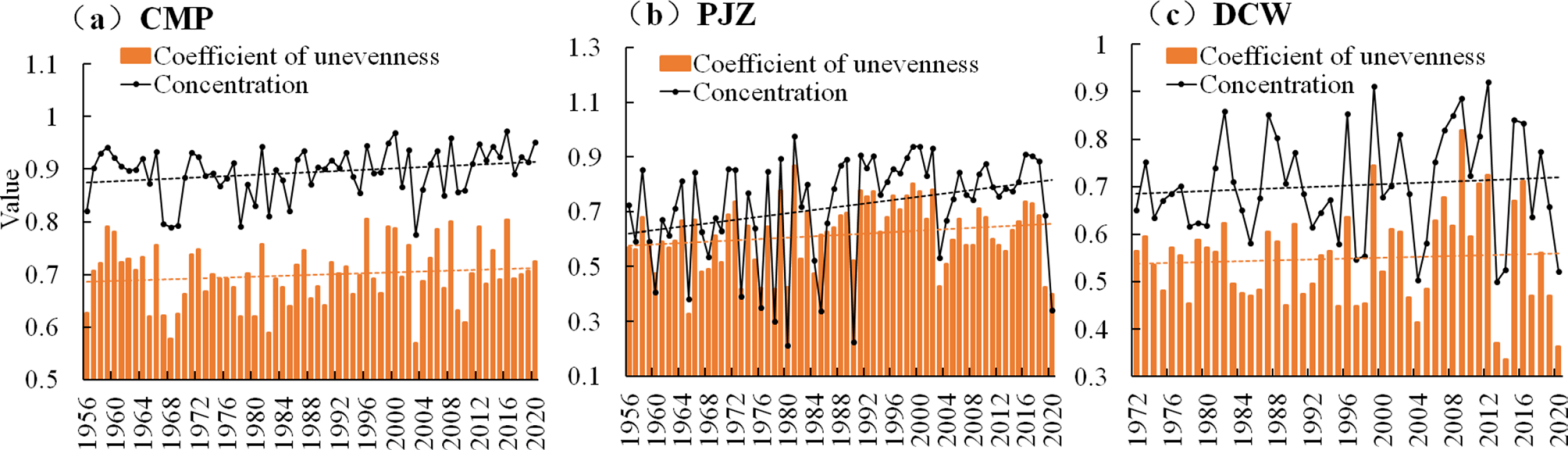
Plot of interannual variability in the coefficient of unevenness and concentration of sand delivery rate.

The non-uniformity coefficient of sediment transport rate of CMP 65a ranges from 0.57 to 0.81 (Fig. 10a), the annual average value is 0.70. The maximum value of 0.81 was recorded in 1996, while the minimum value of 0.57 occurred in 2003. The average annual concentration of sediment transport rate is 0.89, with a maximum of 0.97 in 2016 and a minimum of 0.78 in 2003. This results in a range of 0.20 and a range ratio of 1.25, with concentrations varying between 0.78 and 0.97. For PJZ sediment transport rate 65a, the non-uniformity coefficient varies from 0.33 to 0.87 (Fig. 10b), with an annual average of 0.62. The average annual concentration of this sediment transport rate is 0.72, with a maximum of 0.98 in 1981 and a minimum of 0.21 in 1980. Range value is 0.76, range ratio is 4.56.

The non-uniformity coefficient of DCW sediment transport rate 49a ranges from 0.34 to 0.82 (Fig. 10c), with an annual average of 0.55. The annual average concentration is 0.70, with a maximum of 0.92 in 2012 and a minimum of 0.50 in 2013.

### Spatial variation characteristics of sediment transport rate

**Figure 11:**
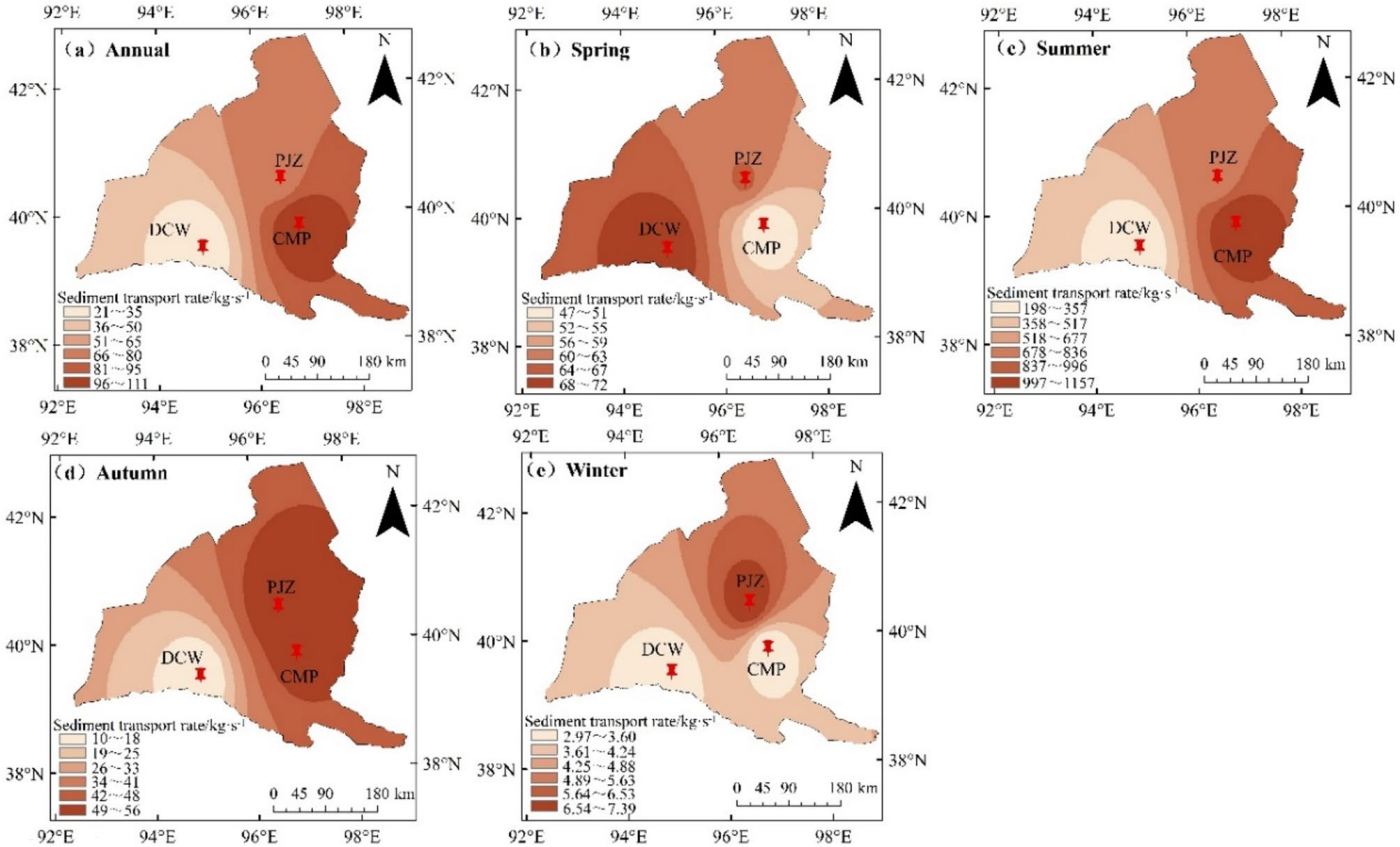
Spatial variation of sediment transport rate in Shule River Basin. Note: The figure made in ARCGIS10.7 (https://desktop.arcgis.com/zh-cn/index.html), the using of natural resources reproduction standard mapping (http://211.159.153.75/index.html). The approval number is GS (2019) 3333, and the boundary of the base map is not modified.

The overall distribution characteristics of sediment transport rate in Shule River Basin are decreasing from east to west (Fig. 11). The annual average sediment transport rates of CMP, PJZ and DCW are 1305.43, 810.06 and 247.80 kg/s, respectively, indicating that CMP is significantly higher than that of other hydrological stations. The average sediment transport rates in spring were 47.05, 64.65 and 71.44 kg/s, respectively, with DCW > PJZ > CMP. The average sediment transport rates in summer were 1203.60, 681.58 and 162.68 kg/s, respectively, showing that CMP was larger than other stations, and the distribution was consistent with the whole year. The average sediment transport rate in autumn and winter varies from 10–56 and 2.9–7.4, respectively, which is manifested as PJZ > CMP > DCW.

## Discussions

### Analysis of the influence of different time scales on the change of sediment transport rate

#### Causes of annual change

**Figure 12:**
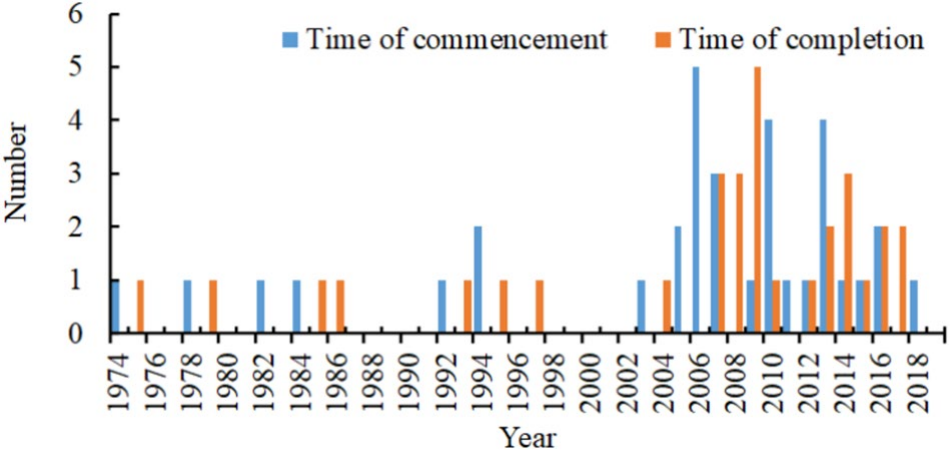
Construction of Danghe hydropower Station during the study period.

1. The calculation results of the sliding average 5a method and linear tendency method in the Shule River Basin, as shown in Table 5, indicate that the sediment transport rate at various stations exhibits distinct annual and seasonal variations. This variation is attributed to significant changes in runoff volume between flood and non-flood seasons, leading to corresponding changes in sediment transport rates. These findings align with previous studies^43^. While PJZ is located downstream of CMP, the sediment transport rate of PJZ is lower than that of CMP. This is due to the fact that the main stream sediment primarily originates from upstream of the outlet pass. Additionally, the water below the outlet pass is significantly impacted by human activities, leading to sediment accumulation and a subsequent decrease in sediment transport rate in the downstream river^44^. The results of Sen's slope estimation method and M–K trend test indicate that the annual, summer, and autumn sediment transport rates of CMP and PJZ are increasing, while the spring and winter sediment transport rates are decreasing. The annual and seasonal variation of DCW also shows a significant upward trend, consistent with previous studies. Human activities, such as soil and water conservation, play a significant role in causing changes in sediment transport rates. In the Shule River Basin, precipitation above the mountain pass is the primary factor influencing sediment levels, while below the pass, factors such as reservoir construction, hydropower stations, diversion projects, and river sand mining are key contributors. Following the years 1994 and 2002, there was a notable increase in the construction of water conservancy projects in Danghe (Fig. 12), causing increased disruptions in the river ecosystem as a consequence of human activities. Consequently, the Dynamic Change of Water (DCW) was impacted by a combination of natural and human-induced factors, leading to slightly differing research findings based on the methodologies employed.
2. The analysis of abrupt changes in the annual mean sediment transport rate within the Schuler River Basin indicates that such changes occurred at three monitoring stations in 1994. The period following 1994 was marked by significant economic development in the basin, which had a notable impact on the ecological environment. The abrupt changes in sediment transport rates align with the annual sediment distribution differences between flood and non-flood seasons in the Shule River. Factors such as the construction of water conservancy projects, reservoir operations, vegetation restoration, and sand mining in river channels played a crucial role in altering sediment transport. To delve deeper into the impact of human activities like water conservancy project construction, large-scale river regulation, and reclamation on sediment transport changes, the time series was divided into pre-1994 and post-1994 periods. The Changma Reservoir project commenced in 1997 and was officially completed in November 2003. The reservoir construction has partially intercepted sediment in the basin. Human activities have led to an increase in surface bare soil, severe soil erosion, and noticeable sand accumulation. In 2003, the Dangcheng Power Station was constructed in the upper reaches of the Danghe River, with plans for 6 additional power stations by 2016. The ongoing productive activities have caused disturbances in the river, leading to fluctuations in sediment transport rates. Additionally, restoration projects for the Shule River and Danghe River began in 2011. The completion of these ecological protection projects has resulted in a decrease in sediment transport rates.
3. There are significant variations in the periodicity of sediment transport rate in the Shule River Basin, with close connections to the analysis of time series, climate variables, and human interventions. The Hurst index effectively identifies trend patterns in time series data, indicating the strength of persistence or anti-persistence in these trends. This study utilizes a Hurst index grading table (Table 6) based on existing literature. Given the continuous changes in sediment transport rate within the basin, sustainability levels are categorized into 5 tiers ranging from weak to strong, each denoted by distinct colors. The Hurst index of sand transport rate in the Shule River Basin (Table 7) is predominantly found in grades I, II, III, and V, exhibiting significant overall variation ranges and sustained intensification differences^45,46^. The evolution trend of sediment transport rates in the Shule River Basin is characterized by a strong and persistent annual sediment transport rate for CMP and DCW, while PJZ exhibits a weaker annual sediment transport rate. The Hurst index analysis reveals that the sediment transport rates at the three stations show a predominance of grade I and grade III distribution frequencies, indicating a mainly strong and persistent trend in sediment transport rates. However, there is also uncertainty in the fluctuation of sediment transport rates under realistic conditions.

**Table 5:** Comparison table of calculation results of different methods for variation trend of sediment transport rate in Shule River Basin.

Stations	Index	Moving average 5a method	Linear inclination method	M–k trend test	Sen’s slope estimation
CMP	All years	Up		Up	Up
	Spring		Down	Down	Down
	Summer		Up	Up	Up
	Autumn		Up	Up	Up
	Winter		Down	Down	Down
PJZ	All years	Up		Up	Up
	Spring		Down	Down	Down
	Summer		Up	Up	Up
	Autumn		Up	Up	Up
	Winter		Down	Down	Down
DCW	All years	Down		Up	Up
	Spring		Down	Up	Up
	Summer		Up	Up	Up
	Autumn		Up	Up	Up
	Winter		Down	Up	Up

**Table 6:**
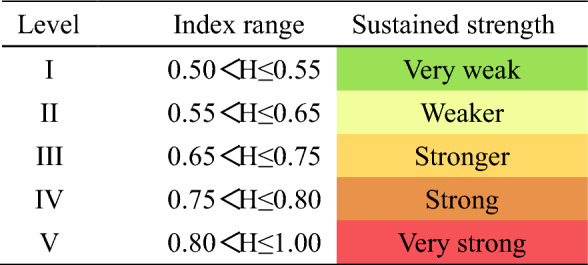
Hurst Index scale.

**Table 7:**
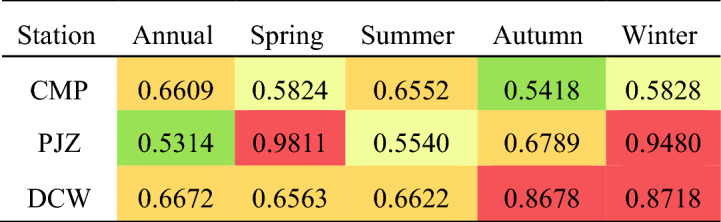
Results of continuous analysis of sediment transport rate throughout the year and four seasons.

#### Reasons for changes within a year

**Figure 13:**
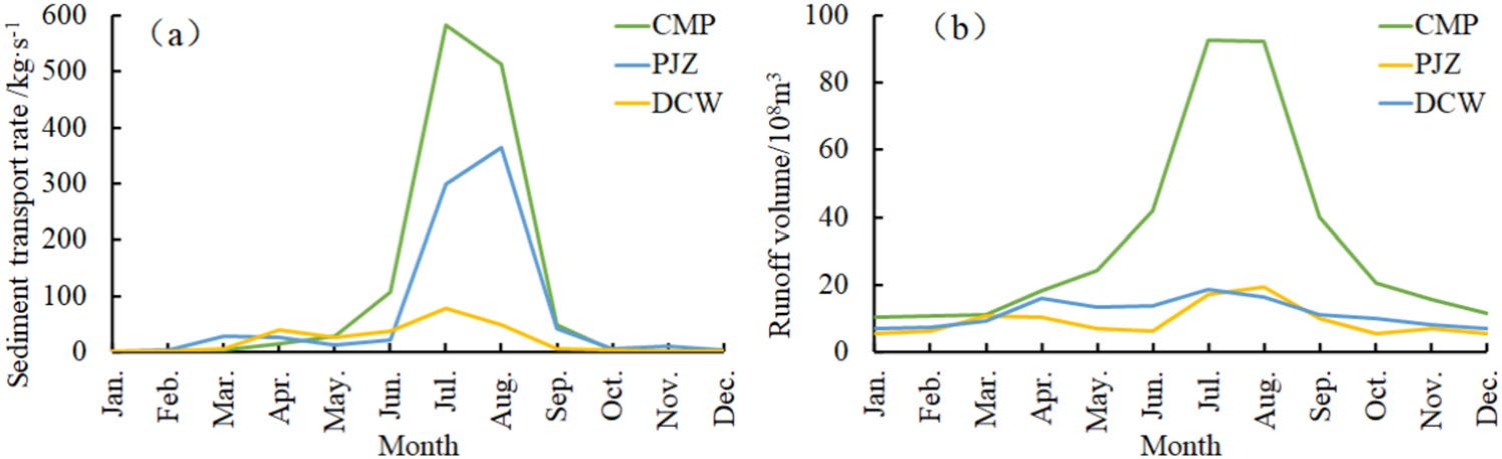
Annual distribution of sediment transport rate and runoff in Shule River Basin.

The annual distribution of sediment transport rates among the three stations in the Shule River Basin is highly uneven. The concentration degree of sediment transport rates is CMP > PJZ > DCW, with sediment transport rates in CMP alternating between July and August, higher in July than in August. PJZ shows alternating concentration periods in July and August, with higher rates in August. The sediment transport rate of the DCW is predominantly observed in July. The annual variation in sediment transport rates within the Shule River Basin is primarily influenced by the annual distribution of runoff and the fluctuations in river sediment content throughout the year^47–49^. The comparison of annual concentration degree and concentration period of annual runoff (Fig. 13b) across the three stations revealed similar change characteristics between the concentration degree of CMP sediment transport rate and runoff concentration. Both show significant concentration in July and August, followed by a rapid decrease in September. However, the concentration degree of sediment transport rate is notably higher than that of runoff concentration, indicating a greater concentration of sediment transport rate throughout the year. The concentration degree and concentration period of DCW sediment transport rate are closely tied to the concentration degree and period of runoff, with both showing clear concentration in July. Nevertheless, the sediment transport rate remains more concentrated over the entire year. The variation in sediment transport rate and runoff concentration of PJZ differs significantly. Analysis of annual sediment transport rates of rivers reveals that while the concentration of PJZ sediment transport rate shows little correlation with changes in runoff concentration, the concentrations of CMP and DCW sediment transport rates are closely linked to runoff concentration fluctuations. This suggests that annual changes in sediment transport rates are primarily influenced by annual variations in runoff, which in turn are impacted by the construction of dams and reservoirs mentioned earlier.

### Analysis of the influence of different spatial scales on the change of sediment transport rate

The spatial variation of sediment transport rate exhibited an increasing trend from west to east, with the annual sediment transport rate at CMP being notably higher than at other hydrographic stations.

The sediment transport in the area is primarily driven by rainstorm floods concentrated during the flood season. CMP is situated in the upper reaches of the Shule River, characterized by a terrain with significant fluctuations, strong river erosion, a high sediment transport rate, and high sediment content. Upon reviewing relevant literature and hydrological data, it was discovered that the Shule River Basin is home to 3 large reservoirs with a combined storage capacity of 4.534 × 10^8^ m^3^ (Table 8), along with 3 medium-sized reservoirs, 76 small reservoirs, and 66 hydropower stations. The soil erosion control area spans 12.879 × 10^4^ hm^2^. Human activities have a significant impact on the sediment transport rate of river basins by altering the sediment content. Research on the influence of human activities on sediment transport rates globally and domestically indicates that these activities primarily modify river sediment content in two main ways. Firstly, changes in vegetation coverage within the river basin can impact soil erosion, subsequently affecting the sediment content of the river^50^. The vegetation coverage rate is influenced by national or regional environmental policies, as well as integrated river basin management. Another factor is the construction of dams and reservoirs within the basin. Upon completion of water storage in DAMS and reservoirs, a significant quantity of sediment tends to accumulate in the area in front of the dam or within the reservoir. This accumulation alters the sediment composition downstream.

**Table 8:** Situation of large-scale reservoir construction in Shule River basin.

River basin	Reservoir name	Reservoir capacity/10^8^ m^3^	Completion time
Shule River	Changma	1.934	2003
Shule River	Shuangta	2.4	1960
Shule River	Chijinxia	0.2	1968

### Analysis of the influence of different driving factors on the change of sediment transport rate

Based on the sediment transport rates and corresponding hydrometeorological data from three major hydrological stations in the Shule River Basin (DCW from 1972–2020, PJZ, and CMP from 1956–2020), the study period was divided into pre and post-1994 for analysis due to abrupt changes observed at all three stations in 1994. Correlation coefficients between sediment transport rate and precipitation, runoff, evaporation, and air temperature were calculated for each station. Scatter plots were then created using Excel2022 to analyze the correlations. The research findings indicated significant variations in correlation coefficients between sediment transport rate and hydrometeorological factors across the different stations.

The sediment transport rates of DCW, PJZ, and CMP show a positive correlation with precipitation and runoff (Fig. 13). This suggests that higher levels of precipitation and runoff lead to increased sediment transport rates. The increase in sediment transport rates can be attributed to the fact that higher precipitation results in more runoff, which in turn washes the surface and rivers, leading to greater sediment accumulation. Prior to 1994, surface evaporation in DCW and CMP was negatively correlated with sediment transport rates, while after 1994, this correlation became positive. Additionally, the temperature in PJZ showed a negative correlation with sediment transport rates before 1994, whereas the temperature in CMP exhibited a positive correlation with sediment transport rates after 1994.

**Figure 14:**
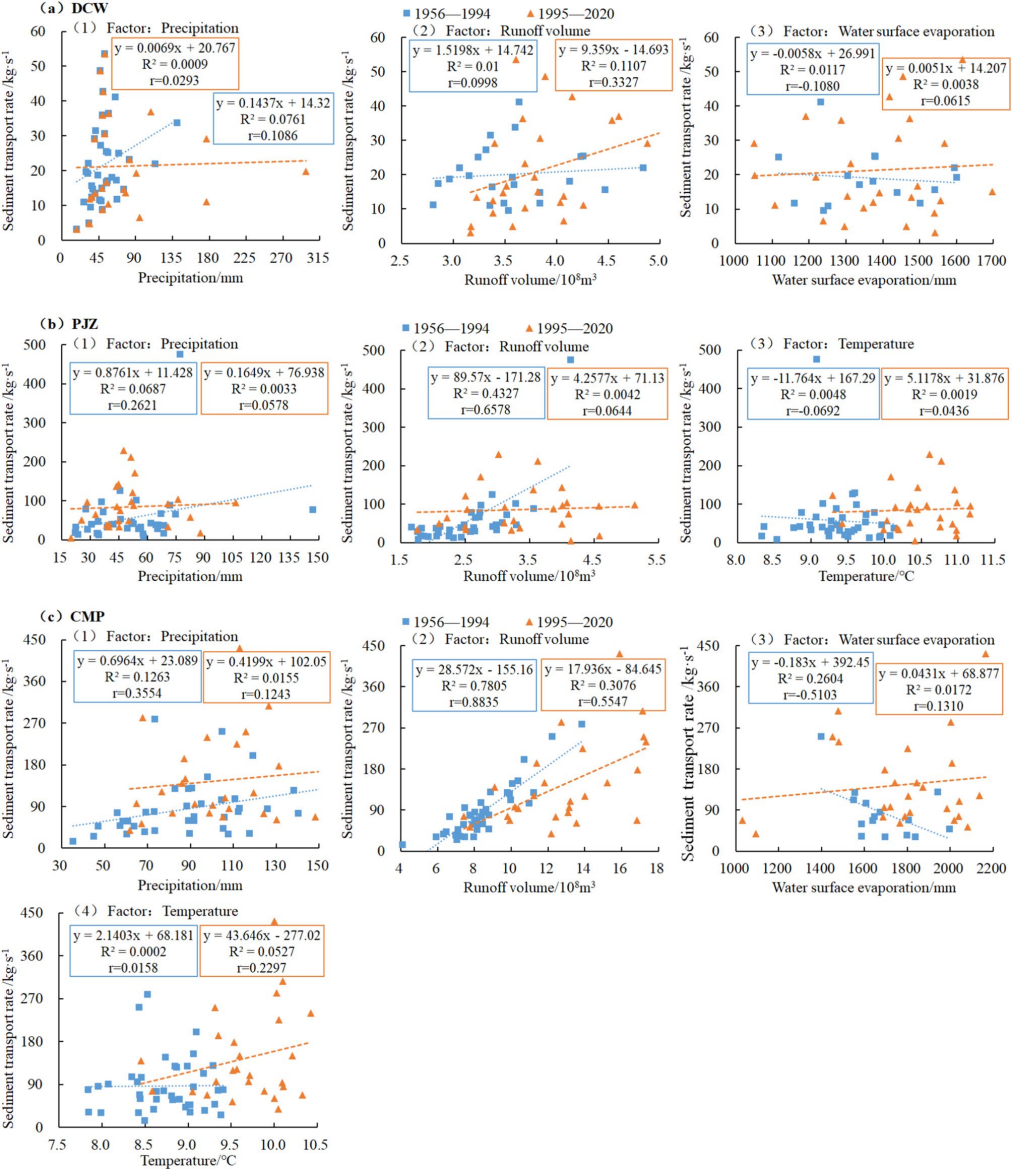
Relationship between average annual precipitation, runoff, evaporation, temperature and average annual sediment transport rate in Shule River Basin.

The DCW sediment transport rate (Fig. 14a) exhibits a weak correlation with precipitation, surface evaporation, and runoff prior to 1994 (|r|< 0.3). Post-1994, runoff also shows a weak correlation with sediment transport rate. The study findings indicate that natural factors of DCW, such as flow velocity and slope direction, positively influence the sediment transport rate. Similarly, the correlation between PJZ sediment transport rate (Fig. 14b) and precipitation, air temperature, and runoff after 1994 (|r|< 0.3) is weak. However, the correlation between PJZ sediment transport rate and runoff before 1994 is considered moderate, with the absolute value of the correlation coefficient ranging between 0.6 and 0.8. These findings suggest that changes in PJZ runoff significantly impacted fluctuations in sediment transport rate before 1994, with this relationship being further influenced by human activities after 1994. Prior to 1994, CMP sediment transport rate exhibited varying correlations with precipitation, runoff, surface evaporation, and air temperature, ranging from low to high to moderate to weak, respectively (Fig. 14c). Following 1994, the sediment transport rate displayed a weak correlation with precipitation, surface evaporation, and air temperature, while exhibiting a moderate correlation with runoff. This suggests that prior to 1994, the variation in sediment transport rate was predominantly influenced by natural factors, whereas post-1994, it was more affected by velocity, slope direction, and human activities.

In the Shule River Basin, variations in CMP sediment transport rates are affected by natural factors like precipitation, runoff, and surface evaporation, as well as human activities such as reservoir and power station construction. On the other hand, the sediment transport rates of PJZ and DCW are primarily influenced by human factors, with the construction of water conservancy facilities and soil and water conservation measures playing key roles in these changes within the basin.

The study found that the runoff of DCW after 1994 was associated with a low sediment transport rate. Results showed that while the variation in DCW runoff did have some impact on sediment transport rate, the effect was minimal. The sediment transport rate of PJZ was moderately correlated with runoff prior to 1994, with the results indicating that changes in PJZ runoff significantly influenced sediment transport rate fluctuations before 1994. However, after 1994, human activities and other factors played a greater role in influencing sediment transport rate variations. Before 1994, the sediment transport rate of CMP showed low, high, and moderate correlations with precipitation, runoff, and surface evaporation, respectively. Post-1994, the sediment transport rate was only moderately correlated with runoff, suggesting that natural factors primarily influenced CMP sediment transport rate variations before 1994, while human activities, as well as natural factors like velocity and slope direction to some extent, were the main influencers after 1994^51,52^.

The results of regression analysis indicate that the significant influencing factors of sediment transport change in the Shule River Basin include precipitation, runoff, evaporation from water surface, and air temperature. Prior to 1994, the order of importance for these factors was runoff > precipitation > evaporation from water surface > air temperature. However, post-1994, the order of significance shifted to runoff volume > surface evaporation > precipitation > temperature. Runoff is identified as the primary factor driving sediment transport, exerting the most direct impact due to its role in soil erosion and sediment transportation. Air temperature influences runoff by affecting evaporation and soil moisture, subsequently impacting sediment transport.

The study results on driving factors in the Shule River Basin indicate significant differences in correlation coefficients of sediment transport rate and hydrometeorological factors across various stations. Natural factors like precipitation, runoff, and water surface evaporation, along with human activities, impact the change in CMP sediment transport rate. Meanwhile, human factors predominantly affect the change in PJZ and DCW sediment transport rates, with water conservancy facilities and soil and water conservation engineering playing crucial roles in altering sediment volume in the basin. Overall, sediment transport in the entire study area demonstrates a fluctuating trend due to the combined effects of natural factors and human activities. Due to the intricate interplay of climatic precipitation and underlying surface conditions in the arid region of northwest China, sediment dynamics are influenced by a multitude of factors including climatic variables and human interventions, leading to significant variations in both temporal and spatial scales. It is recommended to establish additional sediment monitoring stations within the basin to facilitate future investigations into the driving forces behind sediment changes, assess the impact of reservoir operations, and evaluate the effectiveness of ecological engineering initiatives. Subsequent research efforts should focus on enhancing our understanding of the temporal and spatial patterns of sediment transport in response to environmental changes.

## Conclusions

This study investigated the spatial–temporal variation characteristics of sediment transport rate in the Shule River Basin based on annual and seasonal sediment transport data from CMP, PJZ (1956–2020), and DCW (1972–2020). The conclusions drawn are as follows:

1. The annual sediment transport rates of CMP, PJZ and DCW were 12.227 kg/s·(10a)^−1^, 4.318 kg/s·(10a)^−1^ and 0.677 kg·(10a)^−1^, respectively. The sediment transport rates of CMP and PJZ showed an increase during summer and autumn, while decreasing in spring and winter. On the other hand, the sediment transport rates of DCW increased consistently throughout the year and across all four seasons. Each of the three stations displayed distinct seasonality in sediment transport rates, with a notable difference in distribution between flood and non-flood seasons. Human activities, such as changes in runoff and soil and water conservation, were found to be the main factors influencing sediment transport rates.
2. The average annual sediment transport rate of CMP and PJZ experienced significant increases after the 2000s and 2010s, respectively, while DCW exhibited a fluctuating downward trend since the beginning of the twenty-first century. There was a sudden change in sediment transport rates at all three stations around 1994, leading to differences in average annual sediment transport rates before and after this event.
3. The sediment transport rate of the three stations exhibit significant differences in cycle characteristics. The first main cycle of the annual sediment transport rate for CMP, PJZ, and DCW are 51a, 53a, and 29a, respectively. The Hurst index of sediment transport rate shows a relatively wide overall variation range and varying sustained intensities.
4. The distribution indexes of sediment transport rate at the three stations, including non-uniformity coefficient and concentration degree, all exhibited an increasing trend. The annual distribution is highly uneven, with CMP having a higher annual concentration of sediment transport rate compared to PJZ and DCW.
5. Climate change can impact sediment transport rates, influencing overall runoff. The spatial variation of sediment transport at three stations is as follows: CMP, PJZ, and DCW have annual average rates of 1305.43, 810.06, and 247.80 kg/s, respectively. In spring, the rates are 47.05, 64.65, and 71.44 kg/s; in summer, 1203.60, 681.58, and 162.68 kg/s; in autumn and winter, 10–57 kg/s and 2.9–7.5 kg/s, respectively. Overall, CMP shows significantly higher annual sediment transport rates in summer, autumn, and throughout the year compared to the other hydrographic stations.
6. The influence of driving factors on different stations in the Shule River Basin varies. In DCW, there was a low correlation between runoff and sediment transport rate after 1994. The runoff and sediment transport rate of PJZ from 1956 to 1994 showed a moderate correlation. In CMP, there was a high correlation between runoff and sediment transport rate from 1956 to 1994, and a moderate correlation between runoff and evaporation after 1994.

## Data Availability

The datasets used and/or analyzed during the current study are available from the corresponding author on request. The data are not publicly available due to the sheer volume of raw data and the challenges in disseminating them effectively.
